# A LEAP! FORWARD?: DOES PERSONALIZED EXERCISE HELP MAINTAIN COGNITIVE FUNCTION IN OLDER ADULTS?

**DOI:** 10.1093/geroni/igad104.3103

**Published:** 2023-12-21

**Authors:** Valerie Medina, Jeffrey M Burns, James L Vacek, Jonathan Mahnken, Jonathan Clutton, Angela VanSciver, Amber Watts

**Affiliations:** University of Kansas, Lawrence, Kansas, United States; KU Medical Center, Kansas City, Kansas, United States; KU Medical Center, Kansas City, Kansas, United States; KU Medical Center, Kansas City, Kansas, United States; KU Medical Center, Kansas City, Kansas, United States; KU Medical Center, Kansas City, Kansas, United States; University of Kansas, Lawrence, Kansas, United States

## Abstract

Maintaining cognitive function is important to health in older adulthood. Physical activity can decrease the risk of cognitive decline in older individuals. We tested whether LEAP! Rx, a personalized exercise, and lifestyle education program would improve cognition in older adults. We randomized 220 participants (Mean age (SD) = 72 (4.83)), into the program or a control group. We used the NIH Toolbox Cognitive Battery to assess cognition. We analyzed change in cognitive performance from baseline to week 12 using repeated measures ANCOVA to compare the intervention and control groups, adjusting for age, gender, and education. Referral source (self- vs. physician-referred), treatment group (intervention vs. control), and measurement time (baseline vs. week 12) had a significant three-way interaction F(8, 212) = 4.57, p< 0.05. That is, clinician-referred participants in the intervention group improved more (vs. the clinician-referred control group), whereas self-referred control group participants improved more (vs. the self-referred intervention group). Older age, male gender, and lower education predicted poorer verbal learning F(1,218 = 18.79, p< 0.05; F(1, 218) = 28.67, p< 0.05; F(1, 219) = 4.13. Overall, cognitive scores improved more over 12 weeks in the intervention group compared to the control group. Thus, we conclude that personalized exercise programs may enhance or maintain cognitive function in older adults. The differences we observed in physician-referred versus self-referred participants has implications for implementation and scalability of health behavior change interventions, as well as implications for recruitment methods and representation in clinical trials research broadly.

